# Opinion Dynamics Model with Bounded Confidence and the Sleeper Effect

**DOI:** 10.1155/2022/2092757

**Published:** 2022-06-28

**Authors:** Jing Wei, Yuguang Jia, Hengmin Zhu, Xiaojuan Hong, Weidong Huang

**Affiliations:** ^1^School of Management, Nanjing University of Posts and Telecommunications, Nanjing 210003, China; ^2^The Key Base of Philosophy and Social Science Research in Colleges and Universities in Jiangsu Province, Research Center of Information Industry Fusion Innovation and Emergency Management, Nanjing 210003, China

## Abstract

The evolution of opinions is a complex mechanism. The evolution of individual opinions is not only influenced by own and others but also by psychological effects. Therefore, based on the classical bounded confidence model and the sleeper effect, a new opinion evolution model is proposed in this paper. In this new opinion evolution model, we increase the opinions on every step and take into account the “discount opinions.” Since mental states for human are not easy to measure, we assumed three different initial networks and carried out simulation experiments. To verify the rationality on our model, we compared the effects with and without the sleeper effect, different thresholds, and discounting opinion ratio on opinion aggregation and convergence. Finally, we found the sleeper effect can differently affect the convergence of opinions in different opinion environments.

## 1. Introduction

The development of the information society better meets the mass needs of economic, political culture, and so on; meanwhile, the masses of the requirements in public opinion are also growingly increasing. Therefore, the research in public opinion can help the government to supervise and control the polarization phenomenon of public opinion [1], then in favor of national public security. The evolution of opinion has gradually become one of the main research directions of scholars.

Early scholars used discrete opinion dynamics equations to fit the rule of viewpoint change. In 2006, Grabowski and Kosiński [2] creatively used random interactions between molecules in physics to simulate the communication process between people, forming the famous Issing model [2]. With the deepening of research, scholars have proposed Voters model [3–5], Sznajd model [6–8], and so on. Because these models divide the opinions values into independent points, so they are called discrete opinion evolution models. However, the discrete opinion evolution model cannot accurately describe the fuzzy attitude, so scholars put forward the bounded confidence model based on the condition that the opinion change, among which the most classical ones are HK model [9–11] and DW model [12–14].

Some scholars have studied the convergence of bounded confidence models and their modified model. In the study, Schawe et al. considered the heterogeneity confidence level of HK model and found the counterintuitive behavior mechanism in a homogeneous situation in a certain regional stage [15]. Chen et al. [16] concluded that although the convergence characteristics of the DW model can be observed numerically based on reasonable conjecture, it is difficult to analyze technically [16]. In addition, some scholars have also considered the changes in the time when studying the convergence of models [17, 18].

Some scholars combine opinion leaders [19–21], network media [22, 23], information content [24], opinion form [25, 26], and network structure [27, 28] with the bounded confidence model to enrich the application of the bounded confidence model. For example, Chen et al. suggested that leaders' opinions are important in guiding normal individuals to access truth, and that in some cases, small changes in parameters about individual characteristics can lead to large-scale changes in social groups. Zhu et al. [22] believed that media plays a significant role in the evolution of public opinion, so built a dynamic mathematical model of public opinion under the influence of media and analyzed the best opportunity for media to intervene in public opinion. Goddard et al. [29] considered radical population and noisy environment and performed numerical methods to determine the effects of the boundary conditions on opinions. They also found that the no-flux case most faithfully reproduces the underlying mechanisms in the associated deterministic models of Hegselmann and Krause.

To improve the shortcomings that simulations explain models oversimply and defectively, the scholars began to introduce the psychological theories into the opinion dynamics model to strengthen the rationality on their proposed models [30–33]. Li et al. [30] constructed a new opinion evolution equation based on cognitive dissonance theory and studied the influence of bounded confidence and initial connection probability on group opinion. Cheng and Yu [31] considered the influence of group pressure on the evolution of opinions and theoretically proved that all individuals could reach a consensus when facing group pressure in a limited time. Some scholars added social judgement theory, silent spiral, and other theories into the bounded confidence model, which enhanced the reliability and validity of the model evolution results with the addition of psychology.

The remainder of the paper is organized as follows: we review some relevant knowledge about opinion dynamics model and the sleeper effect review in Section 2. In Section 3, we construct a new opinion dynamics model combined with the sleeper effect, which breaks the defect that individuals only communicate with individuals with similar opinion and broadens the scope of interaction of opinions.  Section 4 compares simulation results under different initial distribute on opinions to study the validity and rational on our model. Finally, conclusions and contributions are given in Section 5.

## 2. Preliminary

### 2.1. The Classical DW Model

The DW model was proposed as early as 2004 and belongs to the category of bounded confidence model, which is used to study the formation, evolution, splitting, and convergence of opinions. The classical Deffuant model holds that the population contains *N* individuals, each of which has opinion value *x*_*i*_(*i*=1,2,…, *N*) and *x*_*i*_ ∈ [0,1]. When the difference between the opinions of agent *i* and *j* is less than or equal to the threshold *ε*, *x*_*i*_ will change, otherwise it stays the same. The rules of opinion evolution are as follows:

(1) $$\begin{array}{c} \left\{\begin{array}{cc} x_{i}\left(t+1\right)=x_{i}\left(t\right)+\mu *\left[x_{j}\left(t\right)-x_{i}\left(t\right)\right], & \text{if }\left|x_{i}\left(t\right)-x_{j}\left(t\right)\right|\leq \varepsilon ,\\ x\left(t+1\right)=x_{i}\left(t\right), & \text{if }\left|x_{i}\left(t\right)-x_{j}\left(t\right)\right|>\varepsilon . \end{array}\right. \end{array}$$

The parameter *μ* represents the acceptance of the difference of opinions, generally  *μ* ∈ [0,0.5]. When *μ* is close to 0, it indicates that the agent *i* is not easy to accept to others' opinions. On the contrary, it indicates that the agent *i* is more likely to accept to others' opinions.

### 2.2. The Sleeper Effect

Since the 1930s, with the development of social psychology, scholars' research on attitudes has expanded to multiple disciplines and directions. Among other things, in studies of the temporal effects of persuasion, the researchers found an anomaly: the effects of persuasion sometimes improved over time rather than decreased over time, as was commonly thought.

When Hovland studied the immediate and delayed effects of *The Battle of Britain* on the respondents' attitude change, he found that the effect on attitudes was greater after nine weeks than after five days. The effect of attitude change increases rather than decreases over time [34]. Therefore, Hovland [35] created a new concept “sleeper effect” to describe this phenomenon [35].

Combined with the studies of later scholars, the sleeper effect is defined as follows: “some information is often accompanied by discounted cues (e.g., information claims and sources of low credibility), which will cause the receiver to doubt the validity of the information and inhibit any possible attitude changes by only being exposed to the information; also, when people are presented with persuasive information and discount tips, they tend to be more persuaded over time” [36, 37].

For example, during important elections for political campaigns, undecided voters often see negative ads about a party or candidate. At the end of the AD, they may also notice that the opposing candidate paid for the AD. Presumably, this makes voters question the veracity of the ads and, as a result, they may not be initially convinced. However, even if the source of the AD lacks credibility, voters are more likely to be persuaded later (and ultimately vote against the candidate endorsed by the AD). This shows that the sleeper effect has a subtle influence on the evolution of individual views.

## 3. Modeling Based on Sleeper Effect

Over the past two decades, the world's netizen has grown by the day. Furthermore, for current social platforms, the information recommendation technology based on big data has been adopted widely. The information closing to user preferences will emerge around them, which makes the user get exposure to many opinions which are similar with theirs. At the same time, considering the psychological effect such as group pressure and exponential explosive growth in information, we increase the number of agent interaction per time step which breaks the limitation of interacting with a single agent in the classical model.

Therefore, it is assumed that there exists a bounded confidence threshold *ε*_1_, and *I*(*A*_*i*_, *X*(*t*)) is the trust set of agent *A*_*i*_ at time  *t*, where

(2) $$\begin{array}{c} I\left(A_{i},\;X\left(t\right)\right)=\left\{A_{j}\left\|x_{i}\left(t\right)-x_{j}\left(t\right)\right|\leq \varepsilon_{1}\right\},\;i=1,2,\ldots ,N. \end{array}$$

Based on the classical DW model, the new evolution rule of opinion *x*_*i*_(*t*+1) is defined as

(3) $$\begin{array}{c} x_{i}\left(t+1\right)=x_{i}\left(t\right)+\mu_{i}\sum_{j=1}^{\#I\left(A_{i},\;X\left(t\right)\right)}\left[x_{j}\left(t\right)-x_{i}\left(t\right)\right],\;i=1,2,\ldots ,N. \end{array}$$

#*I*(*A*_*i*_, *X*(*t*)) represents the number of agents in the *I*(*A*_*i*_,  *X*(*t*)), and *μ*_*i*_ represents the acceptance of agent *A*_*i*_ to agents with similar opinions.

In the classical bounded confidence model, the agents usually only interact with individuals with similar views. When the sleeper effect is introduced, the change of agent opinion will also be affected by opinions with discount cues. We assume that there is a threshold *ε*_2_,  *ε*_2_ ∈ (0.5, 1). When |*x*_*i*_(*t*) − *x*_*j*_(*t*)| ≥ *ε*_2_, we call the opinion *x*_*j*_(*t*) of the agent *A*_*j*_ at time  *t* is the “discount opinions” for agent *i*. *S*(*A*_*i*_, *X*(*t*)) is the discount opinions set of agent *A*_*i*_ at time  *t*, where

(4) $$\begin{array}{c} S\left(A_{i},X\left(t\right)\right)=\left\{A_{j}\left\|x_{j}\left(t\right)-x_{i}\left(t\right)\right|\geq \varepsilon_{2}\right\},\;i=1,2,\ldots ,N. \end{array}$$

*S*(*A*_*i*_, *X*(*t*)) represents the number of agents in the *I*(*A*_*i*_,  *X*(*t*)).

**Figure 1:**
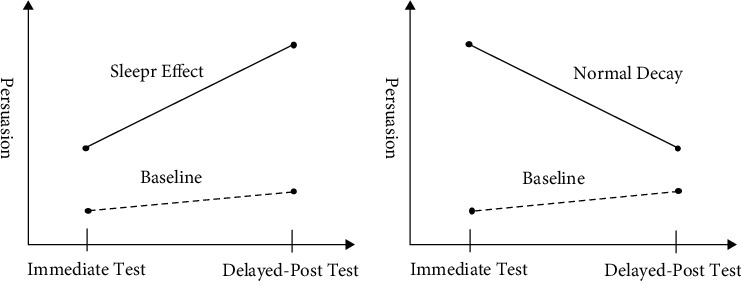
Persuasion under normal decay and sleeper baseline.

Generally, we assume that acceptance of agent is static in research. However, in this paper, considering the characteristics of individual and information, as well as the effect of sleeper effect, we think the acceptance is dynamic. We define *λ*_*i*_(*t*) as the acceptance of agent *i* to “discount opinion” at time *t*. According to Figure 1, agent's acceptance to discount opinions will be affected by the time; that is, the earlier the agent interact with discount opinions, the greater the impact on own. So, we define

(5) $$\begin{array}{c} \omega_{t}\left(T\right)=\frac{t-T}{\sum^{}\left(t-1\right)}. \end{array}$$

Formula (5) represents the relative influence of agent on acceptance of discount opinions at past time *T* (*T* < *t*). For instance, when *t*=30, we simply consider inversely proportional function. In the past time  *T*=1, *T*=2,…,  *T*=29. The influence on acceptance to *λ*_*i*_(*t*) is, respectively, *ω*_30_(1)=29/435,  *ω*_30_(2)=28/435,…,  *ω*_30_(29)=1/435.

In addition, as a kind of information, the influence of opinion is not constant. Regardless of the type of information, its influence will decline over time [3]. Meanwhile, an individual has the characteristics of forgetting, and we define the acceptance of agent to the discount opinions as formula (6), where *α* is the attenuation coefficient:

(6) $$\begin{array}{c} \lambda_{i}\left(T\right)=\lambda_{i}*\omega_{t}\left(T\right)*e^{-\alpha \left(t-T\right)},\;i=1,2,\ldots ,N.\; \end{array}$$

In conclusion, we hold that each agent's opinion will be influenced by its original opinion, its similar opinions, and discount opinions in past. So, *x*_*i*_(*t*+1) will be updated as a weighted aggregation of above opinions, as follows:

(7) $$\begin{array}{c} x_{i}\left(t+1\right)=x_{i}\left(t\right)+\mu_{i}\sum_{A_{j}\mathrm{\epsilon I}\left(A_{i},X\left(t\right)\right)}\left[x_{j}\left(t\right)-x_{i}\left(t\right)\right]+\sum_{T\epsilon \left(1,t-1\right)}\lambda_{i}\left(T\right)\sum_{A_{j}\epsilon S\left(A_{i},X\left(T\right)\right)}\left[x_{j}\left(T\right)-x_{i}\left(T\right)\right],\;i=1,2,\ldots ,N. \end{array}$$

For example, when *t*=4, *α*=0.2, the opinion evolution rule of agent *A*_*i*_ is

(8) $$\begin{array}{c} x_{i}\left(5\right)=x_{i}\left(4\right)+\mu_{i}\sum_{A_{j}\varepsilon I\left(A_{i},X\left(t\right)\right)}\left[x_{j}\left(4\right)-x_{i}\left(4\right)\right]+\lambda_{i}*\frac{1}{1+2+3}*e^{-0.2*1}\sum_{A_{j}\varepsilon S\left(A_{i},X\left(3\right)\right)}\left[x_{j}\left(3\right)-x_{i}\left(3\right)\right]\\ +\lambda_{i}*\frac{2}{1+2+3}*e^{-0.2*2}\sum_{A_{j}\varepsilon S\left(A_{i},X\left(2\right)\right)}\left[x_{j}\left(2\right)-x_{i}\left(2\right)\right]+\lambda_{i}*\frac{3}{1+2+3}*e^{-0.2*3}\sum_{A_{j}\varepsilon S\left(A_{i},X\left(1\right)\right)}\left[x_{j}\left(1\right)-x_{i}\left(1\right)\right]. \end{array}$$

## 4. Simulation Experiments

To compare the influence of sleeper effect on opinion evolution in different scenarios, three typical initial opinion distributions are considered in the simulation experiment: (a) the initial opinions are uniformly distributed in interval [0,1]. (b) The initial opinions are distributed at one extreme partly. In this distribution, some agents have extreme opinions and gather in 0 or 1. (c) The initial opinions are distributed at two extremes partly. In this distribution, some agents have extreme opinions and gather in 0 and 1, respectively. At the same time, for the convenience of expression, we call the agents whose opinion value is 1 as “one extremists,” and the agents whose opinion value is 0 as “zero extremists.” Finally, to ensure the comparability of simulation experiments under different distributions, we set the initial number of agents *N* to 100.

### 4.1. The Initial Opinions Are Uniformly Distributed

**Figure 2:**
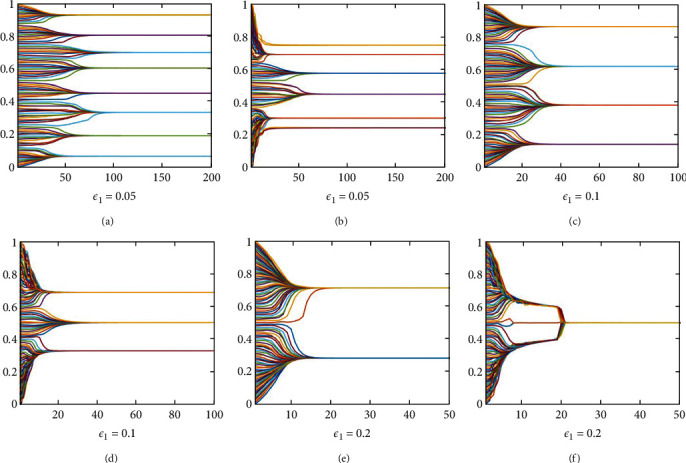
Evolution process of agents' opinions under different *ε*_1_ with or without sleeper effect. (a) *ε*_1_=0.05. (b) *ε*_1_=0.05. (c) *ε*_1_=0.1. (d) *ε*_1_=0.1. (e) *ε*_1_=0.2. (f) *ε*_1_=0.2.

To compare the opinions evolution results with or without sleep effect clearly, we set the same bounded confidence *ε*_1_, respectively, and constant threshold *ε*_2_ to run the simulation according to formulas (3) and (7), which are shown in Figure 2. The left panels Figures 2(a), 2(c), and 2(e) show results using formula (3) where bounded confidence *ε*_1_=0.05, 0.1, 0.2, respectively. The right panels Figures 2(b), 2(d), and 2(f) show results using formula (7) where *ε*_1_=0.05, 0.1, 0.2 , respectively, and threshold *ε*_2_=0.7, and recession coefficient *α*=0.2.

From Figure 2, we can distinctly observe that the number of opinion clusters and time when opinions stay stable will decrease with the increase of bounded confidence *ε*_1_. This indicates that the larger the *ε*_1_ value is, the more tolerant the agent is to different opinion, and the more likely they are to reach social consensus. It is worth noting that this keeps true in our proposed new model. We can also find that the cluster number with the sleeper effect are larger than that without the sleeper effect under the same bounded confidence *ε*_1_. These observations can be explained by the sleeper effect. Under the sleeper effect, opinions in the vicinity of the extreme are subtly drawn in the opposite direction, which coincides with the philosophy that things will develop in the opposite direction when they become extreme.

**Figure 3:**
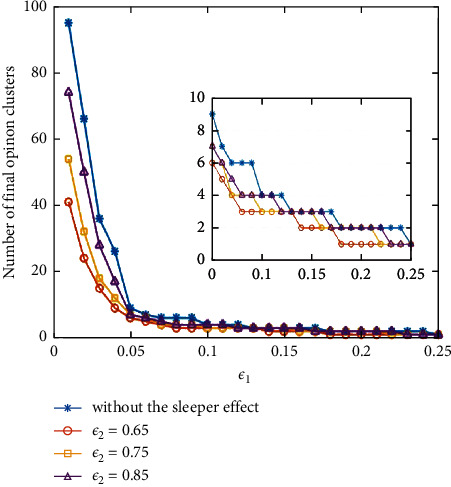
The clusters number under different *ε*_1_ and *ε*_2_.

Next, in another experiments, we set the threshold different *ε*_2_ and control experiment without the sleeper effect. Then, we run the simulation, respectively, to obtain the final cluster number under different bounded confidence *ε*_1_, which are shown in Figure 3. From Figure 3, we can observe that with the increase in bounded confidence *ε*_1_, the final number of opinion clusters will decrease correspondingly, which is consistent with the result in Figure 2. Significantly, when *ε*_1_ ≤ 0.05, the convergence is weak; when *ε*_1_ > 0.05, the convergence is better. It also suggests that a larger *ε*_1_ contributes to consensus.

Furthermore, as shown in Figure 3, with the decrease of threshold *ε*_2_, the number of opinion clusters decreased faster. The observation can be explained by the number of discount opinions. A smaller threshold *ε*_2_  results in agents being exposed to more discount opinions, which makes aggregating easier, especially with a smaller *ε*_1_. In other words, in a society where agents are affected by the sleeper effect, agreement can be reached more easily even in a relatively smaller bounded confidence environment.

### 4.2. The Part of Initial Opinions Is Distributed at One Extreme

In this simulation, we set *r* be the ratio of the extremists, where 0 ≤ *r* ≤ 1. And we assume that initial *N∗r* agents areone extremist, namely, *x*(0)_*i*_=1,  *i*=1,2,…, *N∗r*. The initial opinions of rest agents are distributed uniformly within the interval [0,1], namely, *x*_*i*_(0) ∈ [0,1],  *i*=*N∗r*+1, *N∗r*+2,…, *N*.

#### 4.2.1. The Influences of Bounded Confidence *ε*_1_

**Figure 4:**
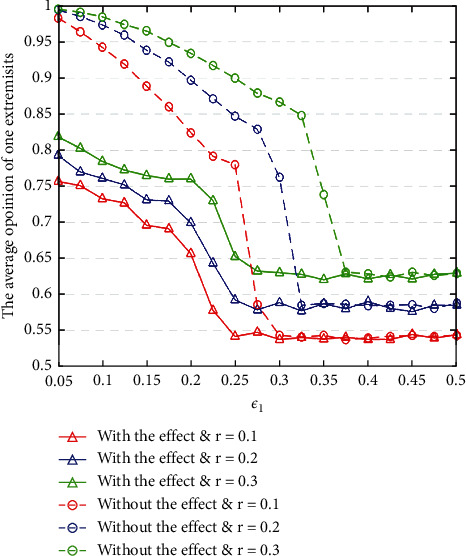
The average stabilized opinions of one extremist under different  *r* and *ε*_1_ with or without the sleeper effect.

Firstly, we set different ratio of the extremists *r*, different *ε*_1_, and the same *ε*_2_ value, then run the simulation 500 times to obtain average stabilized opinions of extremists with or without the sleeper effect, which are shown in Figure 4. From Figure 4, we can observe that average opinions of extremists with the sleeper effect are larger than that without that in the relatively smaller *ε*_1_ under the same initial opinion distribution. While, in the relatively larger *ε*_1_, average opinions of extremists with and without the sleeper effect are consistent within certain range. These observations indicate that, for the extremists, the sleeper effect is more pronounced in changing opinions in a relatively smaller bounded confidence environment.

**Figure 5:**
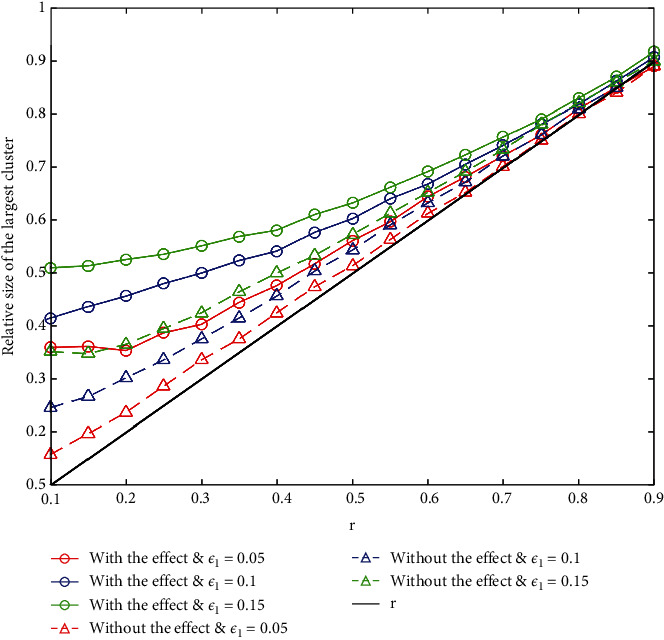
Relative size of the largest cluster under different *ε*_1_ and *r*.

Next, we set different *r* and *ε*_1_, and then run the simulation 500 times to obtain relative size of the largest cluster when all opinions stay stable, which are shown in Figure 5. The relative size is the ratio between the number of agents in the largest final opinion cluster and the size of the agent population.

From Figure 5, we can observe that the relative size of the largest cluster increases with the increase of *r*, but the uprate decreases gradually and approaches to *r* value. It can be explained that the increase of *r* leads to the increase of extremists in the group, which trigger for the increase in the relative size of the largest cluster inevitably. However, when the sleeper effect exists, it enhances the interaction between ordinary agents and extremists, making it easier for most agents to reach a consensus.

#### 4.2.2. The Influences of Threshold *ε*_2_

**Figure 6:**
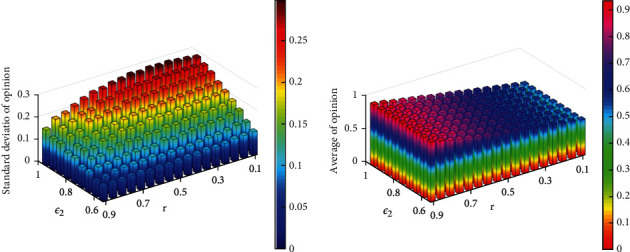
The standard deviation and mean under different *ε*_2_ and *r*.

We set constant bounded confidences *ε*_1_ to ensure the comparability of each experiment group and different *ε*_2_ and *r*. Then, we run the simulation 500 times to obtain the standard deviation and mean under different parameters, which are shown in Figure 6.

From Figure 6, we can observe that with the increase of *r* and the decrease of *ε*_2_, the standard deviation of the opinions decreases gradually; otherwise, the standard deviation gradually increases. Meanwhile, the mean of opinions does not change as *ε*_2_ changes when *r* is constant. It indicates that, the smaller *ε*_2_ is, the stronger the opinion aggregation is, when the initial opinion distribution is the same. Particularly, when *ε*_2_=0.55 and *r*=0.9, the standard deviation is smallest and the mean is largest, which is easy to cause group polarization.

### 4.3. The Parts of Initial Opinions Are Distributed at Two Extremes

In this simulation, we set *r*_0_ and *r*_1_ be the ratios of zero extremist and one extremist, respectively, in the two polar distribution of initial opinions and assume that the number of one extremist is equal to the number of zero extremists, namely, *r*_0_=*r*_1_ and 0 < *r*_0_, *r*_1_ < 0.5. Subsequently, we hold that initial *N∗r*_1_ agents are one extremists, where *x*(0)_*i*_=1,  *i*=1,2,…, *N∗r*_1_, and that initial *N∗r*_0_ agents are zero extremists, where *x*(0)_*i*_=0,  *i*=*N* − *N∗r*_1_+1, *N* − *N∗r*_1_+2,…, *N* − *N∗r*_1_+*N∗r*_0_. The initial opinions of the rest agents are distributed uniformly within the interval [0,1].

#### 4.3.1. The Influences of Bounded Confidence *ε*_1_

**Figure 7:**
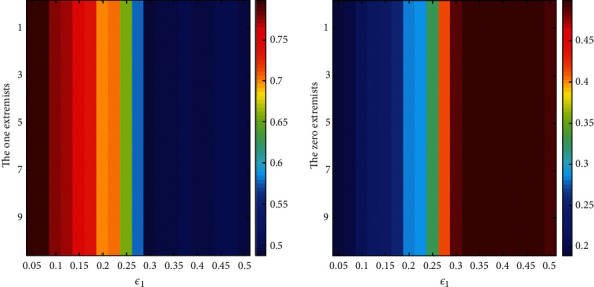
The stabilized opinion values of extremists under different *ε*_1_.

**Figure 8:**
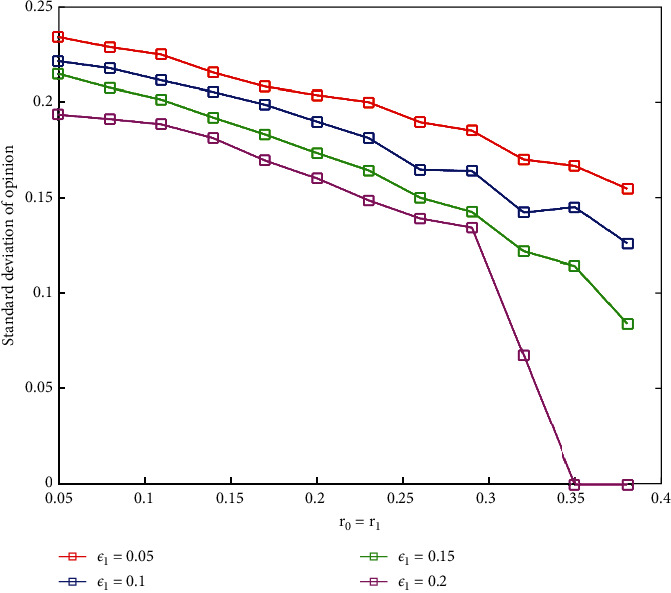
The standard deviation under different *ε*_1_ and *r*_0_(*r*_1_).

First, we set stationary parameters *ε*_2_=0.85 and *r*_0_=*r*_1_=0.1 and then run the simulation 500 times to obtain the average stabilized opinions of extremists under different *ε*_1_, which are shown in Figure 7. From Figure 8, we can observe that the values on opinion for one extremists decrease as the *ε*_1_ increases, while the values on opinion for one extremists increase as the *ε*_1_ increases; however, they keep steady at the 0.5. Compared with Figure 4, it indicates that the speed on convergence for the extremists at two sides is similar with that for the extremists at one side, which is fit to the rule on the classic DW model.

We set different *r*_0_(*r*_1_),  *ε*_1_, and constant threshold *ε*_2_ and then run the simulation 500 times to obtain average standard deviation of all agents' opinions, which are shown in Figure 8. From Figure 8, we can observe that the standard deviation of opinion decreases with the increase of *ε*_1_ when *r*_0_(*r*_1_) is constant. Importantly, when *ε*_1_=0.2 and *r*_0_(*r*_1_)=0.35, the standard deviation of the final opinion drops to 0. It manifests that, although the initial opinions are extreme and scattered, due to the increase of discount opinions, it is still possible to reach an agreement in a relatively small *ε*_1_. In addition, under the sleeper effect, the more extreme the initial opinion distribution is, the more likely it is to form social consensus.

#### 4.3.2. The Influences of Threshold *ε*_2_

**Figure 9:**
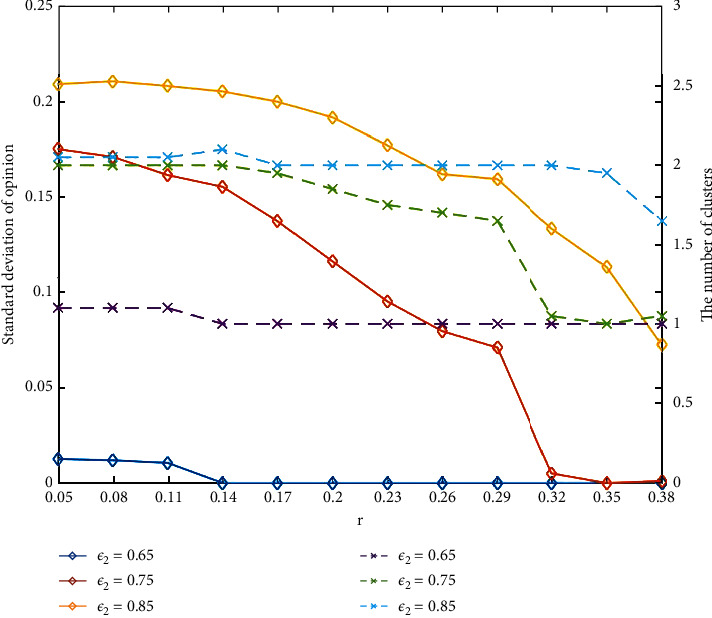
The standard deviation and the number of clusters under different *ε*_2_ and *r*_0_(*r*_1_).

Finally, to study the influences of threshold *ε*_2_, we set the same *ε*_1_ and different *r*, *ε*_2_, and then we run simulation 500 times to obtain average standard deviation and the average number of clusters with different initial opinion distribution, which are shown in Figure 9. The standard deviations are described by solid lines and the number of opinion clusters by dashed lines. From Figure 9, we can observe that the standard deviation decreases as *r*_0_(*r*_1_) increases and *ε*_2_ decreases. It can be explained that, when A is small and the initial opinion distribution is severely extreme, agents will be exposed to a larger number of discount opinions, which leads to the mutual attraction of extremists unconsciously. Thus, agents will reach a social consensus more quickly.

## 5. Conclusions

Based on the classical bounded confidence model and the sleeper effect, a new opinion dynamics model is constructed in this paper. Its main characteristics are as follows: (a) considering the characteristics of individuals in network social interaction, we break the limitation that the classical DW model interacts with a single agent. (b) The sleeper effect is combined with the opinion evolution model, which expands the scope on agents' interaction. (c) In the simulation experiments, we constructed the distribution of different initial opinions and verified the rationality of the model from multiple dimensions.

The main experimental results presented in this paper are as follows: (a) in the uniform distributions of initial opinions, the larger in the bounded confidences is, the less opinion clusters are formed, because of the increase on tolerance for other people. Meanwhile, under the sleeper effect, the relatively more discount opinions can accelerate to achieve opinion convergence. (b) In the one polar distributions of initial opinions, there are more extreme and larger extreme groups as bounded confidences increases. Meanwhile, the tendency on agents' opinions polarization will be accelerated with the increase of discount opinions. (c) In the two polar distributions of initial opinions, larger bounded confidence will yield a larger probability for reaching a consensus. And, unlike an extreme distribution, agency opinions tend to converge to the center as discount opinions increase and generate to reach a strong social consensus.

Although the sleeper effect is difficult to detect, it can be used for reference in the governance of ideas. For example, the “Melatonin,” as a health care product that Chinese people are familiar with, its slogan is deeply rooted in people's hearts. People know that is a marketing strategy for businesses, but they often first choose it as a gift. It inspired that relevant departments need to pay more attention to the number and influence of extremists, because they could exert the profound influence on the ordinary public when controlling public events. The simulation experiments verify the validity of the model, which can provide reference for future information dissemination and public opinion management. In future studies, dynamic networks and dynamic parameters of dynamic with the new dynamical model can be considered to study more complex public opinion.

## Data Availability

The datasets used and/or analyzed during the current study are available from the corresponding author on reasonable request.
